# The Role of Prevention in Reducing the Economic Impact of ME/CFS in Europe: A Report from the Socioeconomics Working Group of the European Network on ME/CFS (EUROMENE)

**DOI:** 10.3390/medicina57040388

**Published:** 2021-04-16

**Authors:** Derek F. H. Pheby, Diana Araja, Uldis Berkis, Elenka Brenna, John Cullinan, Jean-Dominique de Korwin, Lara Gitto, Dyfrig A. Hughes, Rachael M. Hunter, Dominic Trepel, Xia Wang-Steverding

**Affiliations:** 1 Society and Health, Buckinghamshire New University, High Wycombe HP11 2JZ, UK; 2 Department of Dosage Form Technology, Faculty of Pharmacy, Riga Stradins University, Dzirciema Street 16, LV-1007 Riga, Latvia; Diana.Araja@rsu.lv; 3 Institute of Microbiology and Virology, Riga Stradins University, Dzirciema Street 16, LV-1007 Riga, Latvia; Uldis.Berkis@rsu.lv; 4 Department of Economics and Finance, Università Cattolica del Sacro Cuore, Largo Agostino Gemelli 1, 20123 Milan, Italy; elenka.brenna@unicatt.it; 5 School of Business & Economics, National University of Ireland Galway, University Road, H91 TK33 Galway, Ireland; john.cullinan@nuigalway.ie; 6 Internal Medicine Department, University of Lorraine, 34, Cours Léopold CS 25233, F-54052 Nancy, France; jean-dominique.dekorwin@univ-lorraine.fr; 7 University Hospital of Nancy, Rue du Morvan, 54511 Vandoeuvre-Les-Nancy, France; 8 Department of Economics, University of Messina, Piazza Pugliatti 1, 98122 Messina, Italy; lara.gitto@unime.it; 9 Centre for Health Economics & Medicines Evaluation, Bangor University, Bangor LL57 2PZ, UK; d.a.hughes@bangor.ac.uk; 10 Department of Primary Care and Population Health, Royal Free Medical School, University College London, London NW3 2PF, UK; r.hunter@ucl.ac.uk; 11 Global Brain Health Institute, School of Medicine, Trinity College Dublin, College Green, D02 PN40 Dublin, Ireland; trepeld@tcd.ie; 12 Warwick Medical School, University of Warwick, Coventry CV4 7AL, UK; xiasteverding@gmail.com

**Keywords:** prevention, economic impact, chronic fatigue syndrome, myalgic encephalomyelitis, ME/CFS

## Abstract

This report addresses the extent to which there may be scope for preventive programmes for Myalgic Encephalomyelitis/Chronic Fatigue Syndrome (ME/CFS), and, if so, what economic benefits may accrue from the implementation of such programmes. We consider the economic case for prevention programmes, whether there is scope for preventive programmes for ME/CFS, and what are the health and economic benefits to be derived from the implementation of such programmes. We conclude that there is little scope for primary prevention programmes, given that ME/CFS is attributable to a combination of host and environmental risk factors, with host factors appearing to be most prominent, and that there are few identified modifiable risk factors that could be the focus of such programmes. The exception is in the use of agricultural chemicals, particularly organophosphates, where there is scope for intervention, and where Europe-wide programmes of health education to encourage safe use would be beneficial. There is a need for more research on risk factors for ME/CFS to establish a basis for the development of primary prevention programmes, particularly in respect of occupational risk factors. Secondary prevention offers the greatest scope for intervention, to minimise diagnostic delays associated with prolonged illness, increased severity, and increased costs.

## 1. Introduction

Myalgic Encephalomyelitis/Chronic Fatigue Syndrome (ME/CFS) is a complex, serious, multi-system disorder, which is very disabling, with marked diminutions in function in quality of life. Its symptoms include severe fatigue, which is disabling and not improved by rest, and in particular, post-exertional malaise. Other symptoms include sleep disturbance, muscle pain, and cognitive dysfunction [1,2,3,4]. Symptoms, many of which are autonomic in nature, persist for at least six months. There is marked variation in the severity, symptoms, and clinical course of the disease. About three quarters of all patients are female. It occurs in all age groups, but most frequently arises in the 20 to 50 age group [5,6,7]. There may be around two million people with ME/CFS throughout Europe.

The European Network on ME/CFS (EUROMENE) was created to facilitate collaborative research, through working groups on epidemiology, biomarkers, and diagnostic criteria, clinical research, and socioeconomics, Europe-wide, to meet substantial gaps in scientific knowledge. Researchers from twenty-two countries now participate in the network. Working Group 3 (socioeconomics) focuses on the economic and social aspects of ME/CFS, with the objective of estimating the societal burden of ME/CFS.

UK experience suggests that the total cost of ME/CFS in Europe, including direct and indirect healthcare and other costs and productivity losses, may be in the region of €40 billion per annum [8], so even a 1% reduction achieved through programmes of prevention would be a substantial sum, though would need to be compared to the costs of such programmes. This report addresses the extent to which there may be scope for preventive programmes for ME/CFS, and what economic benefits may accrue therefrom.

## 2. The Economic Case for Prevention

There is evidence showing that many preventive programmes represent value for money [9] and that, therefore, there is a strong economic case for implementing them. Such programmes include, for example, targeted supervised tooth brushing and smoking cessation services [10]. Investments in prevention can produce value in terms of reduced healthcare spending, increased productivity, and improved quality of life, particularly when directed at chronic diseases that are major drivers of healthcare costs [11,12]. There are also benefits, in terms of both health and economic consequences of illness, from programmes that are effective, either in preventing illness or in treating it at an early stage, and there is empirical evidence to support this for certain conditions, such as colorectal cancer [13]. 

Thus, in many cases, there are numerous good reasons to invest in a well-defined package of preventive services that are recognised as effective in preventing disease and offer good economic value. The economic case can be demonstrated by cost-effectiveness or cost-utility analyses and/or the calculation of social return on investment (a quasi-cost-benefit analysis), or, where applicable, by cost-minimisation for two or more equivalent services. A review of economic evaluations of public health (PH) interventions assessed by the National Institute for Health and Care Excellence (NICE) found that three-quarters of preventative interventions were cost-effective at a threshold of £20,000 per quality-adjusted life year (QALY) [9]. 

There is evidence indicating that health promotion and primary prevention programmes are cost-effective [14,15], especially when the role of the recipients is passive, as in immunisation programmes, or when the programme is designed to deliver a public good to a whole community, such as fluoridation [16]. In the context of heart disease, as one example, and based on 19 economic evaluations informed by 15 randomised controlled trials, exercise therapy is cost-effective in patients with coronary heart disease, chronic heart failure, intermittent claudication, or with a body mass index (BMI) ≥ 25 kg/m^2^ [17]. Treatments for heart disease are less cost-effective, with the majority of interventions (pharmacological and non-pharmacological) for heart failure associated with incremental cost-effectiveness ratios exceeding USD30,000 per QALY gained [18]. Preventive care, particularly for chronic diseases, can help patients and reduce costs and impacts on economic activity [19]. A study of the impact on healthcare utilisation and expenditure trends of a programme of prevention through behaviour modification found that a primary care model based on the doctor–patient relationship can have a positive impact in improving health, reducing the prevalence of chronic disease and disability, and reducing expenditure [20]. This is confirmed by a Report of the Surgeon General, which concluded that a water fluoridation programme, coupled with other dental initiatives, would improve dental health and cut costs [21]. Another review concluded that there was indeed potential for preventive services to delay or avoid distressing medical conditions that are expensive to treat [22]. Preventive care, particularly for chronic diseases, can help patients and reduce costs and impacts on economic activity [23].

## 3. Impediments to Prevention

A major challenge to successful implementation of programmes of prevention and demonstration of its economic value lies in the innate conservatism of people, and their unwillingness to change behaviour, as well as reticence when it comes to paying for such programmes [24], particularly as they require both a long-term view and intersectoral cooperation, and it can take many years for benefits of prevention to emerge [25]. For example, there is a significant gap in the availability of full economic evaluation studies focused on primary prevention of mental health problems among the elderly, and some patients do not appreciate the benefits of preventive programmes [14]. The evidence base regarding prevention programmes is very limited. In addition, the empirical evidence on individual prevention activities is rarely precise or definitive and there is a lack of high-quality studies. The economic benefits diffuse and appear abstract, and it is not always clear which individuals benefit [22]. In some cases, prevention (e.g., fitness, organic food, and clothing) can cause a prohibitive burden to individual and family budgets. 

## 4. The Content of Prevention

Prevention may be primary, secondary, or tertiary. Primary prevention is designed to stop the onset of disease, often through behaviour modification, while secondary prevention consists of early detection when the disease is asymptomatic, in order to ‘nip it in the bud’. Tertiary prevention is designed to mitigate the consequences of disease through disability limitation and rehabilitation. All three have the potential to reduce the costs of disease [11,24]. Prevention should address the causes of illness, be they social, economic, or environmental, including housing, education, and employment [25]. A focus on health behaviour and environmental and occupational risks is directed towards the main causes of preventable ill health, and important factors to consider in developing prevention programmes include lifestyle, social and community influences, living and working conditions, as well as socioeconomic, cultural, and environmental circumstances [26]. 

## 5. Evaluation of Prevention

Economic efficiency does not imply that cost should be minimised, or benefit maximised, but rather that cost be compared with benefit, and that net health benefits (the incremental cost divided by the opportunity cost threshold) be maximised [24]. The focus of investigation should be to determine whether the benefits accruing for the minority who benefit from a preventive intervention offset the costs (that is, the health benefits foregone) to the population as a whole. 

The studies required to support evidence-based decisions on funding preventive programmes include effectiveness studies, simulation modelling, and economic evaluations [11]. In evaluating prevention programmes, aspects to consider include long-term impacts, non-health and non-monetary impacts, differential impacts across groups, and time preference [27]. Methodologically robust economic evaluations are needed to support decision-making in the allocation of healthcare resources, but especially in the context of prevention, where there are significant uncertainties in determining effectiveness, challenges in the measurement and valuation of outcomes, and often a lack of consideration of inter-sectoral costs, consequences, and equity implications [14,25].

There is a variety of possible approaches to evaluating the health and economic impacts of preventive programmes. Some are of more use to decision makers than others, particularly where they cover a long time-span [21]. Interventions for the prevention of chronic non-communicable diseases (NCDs) and certain types of injuries mainly address programmes designed to modify health-related behaviours and their interaction with environmental influences [28]. Research conducted in the UK since the 1970s stressed the relationship between socioeconomic position and health [26]. The World Health Organization (WHO) Commission on the Social Determinants of Health worked on the basis of a conceptual framework in which two main groups of determinants were identified, structural (e.g., socioeconomic and political contexts, social structures, and socioeconomic position) and intermediary factors (e.g., biological, behavioural, health system and psychosocial factors, living and working conditions) [29].

There is a need to elucidate the nature and extent of the evidence that demonstrates cost-effectiveness of disease and injury prevention programmes and clinical prevention services [11]. Estimating the cost-effectiveness of prevention, generally, is problematic, because such an evaluation may combine interventions of proven effectiveness with others—the effectiveness of which is less certain [23]. Recent reviews of economic evaluations of prevention programmes highlight the methodological limitations and challenges [9,25]. The choice of discount rate, as one example, to account for time preference, can impact significantly on the cost-effectiveness of prevention programmes, as even large future health benefits may result in low net present value.

In considering approaches to evaluation, it is necessary to consider the extent to which modelling methods could be used to project the clinical and spending impact of prevention programmes and whether wider impacts on employment should be taken into account. There is also a need to determine appropriate time horizons for evaluations, to consider how health benefits, including health-related quality of life, should be measured, and the extent to which it is possible to evaluate prevention programmes using traditional economic models [21,29].

Methods for quantifying the (social) return on investment of a proposed prevention programme are gaining popularity. These are consistent, in the UK, with the National Institute for Health and Care Excellence public health guidance, which comments on the appropriateness of cost-benefit analysis for public health programmes. Social return on investment analyses incorporate considerations of effectiveness and its time period, as well as of cost and perspective (i.e., which costs and benefits are included in the analysis) [30,31]. As public health has impacts extending beyond health alone, a broader perspective is often warranted. The pertinent question for prevention is whether it offers good value, in terms of return on investment, bearing in mind that addressing a single risk factor can impact on a broad range of conditions, and that the long-time horizon creates an opportunity for the compounding of health benefits [23].

Taking into account the above considerations, two main questions should be addressed: first, as to whether there is scope for preventive programmes for ME/CFS, and secondly, if so, whether there are health and economic benefits to be derived from the implementation of such programmes [32]. The answer to the first question depends on whether there are risk factors for ME/CFS which are capable of modification by means of such programmes, and this is considered next.

## 6. Risk Factors for ME/CFS

Although the exact pathogenesis of ME/CFS is still unknown, the most plausible hypothesis is that it is a complex multifactorial syndrome in which immunological and environmental factors play a crucial role [33,34].

## 7. Infections

Viral infections are involved in the aetiology of most cases of ME/CFS [35,36]. Various viral illnesses have been implicated, including for example the Epstein–Barr virus [37,38,39,40], and various sites of infection, including gastrointestinal infections [41]. Whether or not a viral infection creates a risk of ME/CFS depends on a number of parameters, including virus burden, strain, patterns of replication, and life cycle [42]. Cases may be epidemic or sporadic, with epidemic cases appearing to have a better prognosis [43].

Other infections which have been implicated as causes of ME/CFS include the Ross River virus and Coxiella burnetti [36]. Infections studied which have not been shown to cause ME/CFS include human herpesvirus 6, enterovirus, rubella, Candida albicans, bornaviruses, mycoplasma, and human immunodeficiency virus (HIV). An increase in the titre of anti-HHV-6 IgG and IgM antibodies in the sera of CFS patients has been demonstrated in comparison with a control population, but this was unspecific, with increases also in antibodies to other viruses, so this may simply reflect underlying immune dysfunction [44].

## 8. Immunological Factors

In addition, ME/CFS has some features in common with autoimmune illnesses and several studies have identified immunologic biomarkers [33]. Thus, both are more common in women and demonstrate increased inflammation. Other ways in which the immune system might contribute to ME/CFS include production of cytokines affecting the body’s ability to respond to stress, low-functioning natural killer (NK) cells, and differences in markers of T-cell activation. Physical or emotional stress causing derangement of the hypothalamic-pituitary-adrenal axis (HPA axis), leading to low levels of cortisol, may thus lead to an increase in inflammation and chronic activation of the immune system. Finally, possible causative factors include immune suppression, increased intestinal permeability, impaired mitochondrial performance, changes in energy production, and a possible genetic link [34,40].

## 9. Occupational Exposures

Most of the concern about chemical exposures as a possible cause of ME/CFS centres on the agricultural use of organophosphates (OPs) and, to a lesser extent, of organochlorines. Fatigue syndromes may be secondary to occupational exposures to organochlorine or organophosphate compounds [45]. Fernández-Solà et al. [46] described a series of twenty-six patients, nine of whom were exposed to organophosphates alone, who developed chronic fatigue following insecticide exposure. Thamaz et al. [47] observed a dose–response relationship between chronic fatigue scores and levels of exposure to organophosphate pesticides.

The EU’s Scientific Steering Committee reviewed the role of organophosphates as agricultural insecticides, used to control arthropod pests, including parasites, such as grub, horn fly, and other cattle exoparasites. It did not consider, however, the possible role of organophosphate exposure as a risk factor for ME/CFS, as their concern was the cause of bovine spongiform encephalopathy (BSE), in respect of which they concluded that there was no evidence to support the hypothesis that organophosphate exposure might be involved [48].

UK press reports assert involvement of organophosphates in the development of ME/CFS, and the risk to highly exposed agricultural workers cannot be disregarded [49]. A study of reports to the UK Veterinary Medicines Directorate of ill health attributed to pesticide exposure among agricultural workers found that ME/CFS-like symptoms were frequently mentioned, and questionnaire responses indicated an association with organophosphate exposure [47]. It appears that the major hazards of pesticide use are poisonings associated with exposure of operators as a result of misuse. This is supported by a study supported by the UK Health and Safety Executive, in which a comparison of 146 sheep dippers exposed to OPs and 143 non-exposed controls (quarry workers) found significant differences between the groups in various neuropsychological tests, such as simple reaction time, symbol-digit substitution, and syntactic reasoning, and also on neurological examination and the General Health Questionnaire. There were no observable differences on tests of memory or psychomotor function. There was evidence of sensory neuropathy of hands and feet among the sheep dippers. The authors concluded that “although the effects identified are not severe, the results of the investigation suggest that further efforts should be made to reduce exposure to organophosphates in terms of identifying the most appropriate protective clothing and dipping equipment and encouraging its use” [50].

Another study found that patients with a fatigue syndrome following organophosphate exposure manifested some differences in symptoms compared with sporadic cases of ME/CFS [51], but both groups conformed to the CDC-94 (Fukuda) case definition [52]. This is confirmed by a study comparing patients with Gulf War syndrome (GWS), ME/CFS, and the fatigue syndrome associated with organophosphate exposure, which found many similarities between the three conditions, but only patients with ME/CFS manifested peripheral cholinergic abnormalities in vascular endothelium, perhaps indicating a different aetiology [52]. Similarly, a study comparing agricultural workers who had been exposed to organophosphates with ME/CFS patients found that the two groups were identical in terms of mode of onset of illness, symptoms, and the results of neuroendocrine studies [53]. Kennedy et al. [51] described patients meeting the diagnostic criteria for CFS/ME following exposure to OPs. Another study compared forearm skin blood flow responses to iontophoresis of acetylcholine that were measured using laser Doppler imaging in patients with ME/CFS, GWS, illness following organophosphate exposure, and matched healthy controls. The acetylcholine response was higher in patients with CFS than in controls, but normal in GWS patients and those exposed to organophosphates, which may suggest aetiological differences [42]. Since ME/CFS is a syndrome, defined by its clinical features rather than underlying pathology [52], it is reasonable to regard the illness which may be a long-term outcome of OP exposure as ME/CFS, since the two conditions have many clinical features in common [53]. This is underlined by another study, which found that similar reproducible abnormalities of gene expression were found in ME/CFS patients and in patients following OP exposure [54].

Various studies have identified a range of long-term neurological abnormalities following OP exposure. These include significantly impaired performance in neuro-behavioural tests and peripheral neuropathy, with impaired memory and concentration, depressed mood [55], delayed neuropathy characterised by weakness or paralysis, and paraesthesia of the extremities, an intermediate syndrome muscular weakness, predominantly involving muscles of the face, neck, and limbs, with cranial nerve palsies and depressed reflexes. These may be related to neuromuscular transmission dysfunction [56], prolonged cognitive processing of visual stimuli [57], and neurocognitive, fibromyalgic, and chronic fatigue manifestations [45]. Acute OP poisoning due to acetylcholinesterase inhibition can lead to permanent disability or delayed peripheral neuropathy. Long-term low-dose effects are not necessarily due to acetylcholinesterase inhibition, however, but may indicate targeting of brain proteins [58]. The long-term consequences of OP exposure observed in humans are also apparent in animal experiments. Thus, repeated exposures of rats to two OPs (chlorpyrifos and diisopropylfluorophosphate) in low doses may lead to chronic deficits in spatial learning and memory [59].

Organochlorines have also been implicated in the development of fatigue syndromes. One study found that patients with unexplained, persistent fatigue had higher levels of DDE (1,1-dichloro-2,2-bis (p-chlorophenyl) ethane—an organochlorine) compared with controls [60]. A study of chlorinated hydrocarbon levels in patients with chronic fatigue syndrome concluded that organochlorines may indeed be involved in the aetiology of ME/CFS [61], and it could be that this involvement of such environmental chemicals is in combination with genetic factors [62]. There have been reports of an outbreak of ME/CFS in Nevada at the same time as an increased incidence of non-Hodgkin’s lymphoma [63,64]. A causal relationship has been suggested [65], but both conditions may be attributable to exposure to agrichemicals, particularly organochlorines [66]. 

In conclusion, most of the studies considered were not population-based, had small sample sizes, or achieved very small response rates, and variations in diagnostic criteria make it difficult to draw general conclusions. A review of the research literature on the role of chemical exposures in the aetiology of ME/CFS concluded that the evidence of possible associations was inconclusive, so more research is needed [67]. However, there are sufficient pointers to conclude that, in respect of OPs, there is sufficient reason to at least adopt a precautionary principle and minimise exposure as far as possible.

## 10. Psychological Factors

Much of the research on risk factors has focused on psychology. Psychological risk factors reported include perfectionism, self-sacrificial tendencies, unhelpful beliefs about emotions, and perceived stress [68], personality disorders, and childhood traumatic experiences [69]. Other psychosocial risk factors proposed include functional somatic syndromes [70], cultural factors [71], other conditions labelled as somatisation disorders such as irritable bowel syndrome [72], socioeconomic deprivation [73], maladaptive personality and personality disorders [74], premorbid stress [75], premorbid distress and depression [76], maternal overprotection [77], and childhood trauma [78,79]. Membership of minority ethnic groups has been identified as another possible risk factor for ME/CFS. However, this may be associated with higher levels of anxiety, depression, physical inactivity, social strain, and lack of social support, rather than being part of an ethnic minority per se [80]. Psychiatric disorders, or shared risk factors for psychiatric disorders, it is asserted may have an aetiological role in some cases of CFS/ME [81], but the evidence for this and the other psychological factors reported here, is equivocal, to say the least.

## 11. Children and Adolescents

In children and adolescents, identified risk factors include family adversity [82], maternal anxiety, or depression [83]. It is more common in those who are socially deprived [84], and also among adolescents who experience anxiety and decreased physical activity [85]. However, other authors have found no relationship between childhood trauma and ME/CFS [86], and much of the evidence for psychosocial risk factors for ME/CFS is conjectural and unconfirmed. A systematic scoping review failed to reveal definitive evidence of risk factors for ME/CFS [87]. Another study failed to find any association between maternal or child psychological distress, academic ability, parental illness, atopy, or birth order and lifetime risk of CFS/ME, which was increased by sedentary behaviour [88]. Another study found physical factors such as disability and fatigue to be more prominent as risk factors for ME/CFS than psychosocial factors such as stress and coping [89]. The studies listed above for the most part identified associations rather than causal relationships, and Hickie et al. concluded that psychological disturbance was likely to be a consequence of ME/CFS, rather than a risk factor for it [90].

## 12. Other Possible Risk Factors

Other possible risk factors have also been suggested but remain unconfirmed. Thus, the risk of ME/CFS is increased if a close family member also has the illness, suggesting a role for genetic factors [91,92]. A questionnaire-based study found that the prevalence of CFS was higher in genetically unrelated household contacts and in non-resident genetic relatives than in the community, indicating that both household contact and genetic relationship are risk factors for CFS [93]. Other proposed factors include female gender, age, previous exposure to stress or toxins, occupational exposures, and infectious diseases, poorer health status [94], gynaecological conditions and surgery [95], ethnic minority status [96], and premorbid persistent unexplained severe fatigue [97,98]. The mechanism through which such risk factors take effect could be oxidative stress [99], while the increased risk of ME/CFS due to profound inactivity, deconditioning, or sleep abnormalities may be mediated via neuroendocrine dysregulation [100].

Two reports from the UK ME/CFS Biobank confirmed that little was known about risk factors for ME/CFS. A cross-sectional study of participants assessed the prevalence of cognitive and sleep symptoms in ME/CFS patients, in comparison with MS patients also participating in the Biobank. Cognitive symptoms included problems with short-term memory, attention, and executive function. Sleep symptoms included unrefreshing sleep and poor quality or inadequate duration of sleep. Such problems were more prevalent in the ME/CFS group than among the MS patients. Older ME/CFS patients (i.e., over 50) were much more likely to experience severe symptoms than younger ones (less than 30) (Odds ratio (OR) 3.23, *p* = 0.031). Severe symptoms were much more common among smokers and those with household incomes below £15,000 per year [101].

A further report found that a previous history of frequent infections, including colds and influenza, were the factors most strongly associated with a higher risk of ME/CFS compared to healthy controls. Other factors were being single, having lower income, and a family history of anxiety. Lower age at onset was associated with more severe disease, as also was a family history of neurological illness, which suggests that genetic and environmental factors may be involved. However, the authors concluded that there was little consistency in published reports [102].

This conclusion was borne out by a recent systematic scoping review of causal factors for CFS/ME. This examined 1161 studies published between 1979 and June 2019. Most were case-control studies, with under 100 participants. Potential factors studied were many and varied and ranged from environmental through to genetic factors. The categories of potential factors most frequently studied were immunological, psychological/psychosocial, socioeconomic, infections, and neuroendocrinal/hormonal/metabolic, with the greatest variety of possible risk factors being examined in the infections category. Studies of viruses predominated, particularly the Epstein–Barr virus, human herpes virus, and xenotropic murine leukaemia-related virus. No one possible causal factor was dominant, indicating much uncertainty in the field. The authors concluded that the quality of the evidence was too low to draw conclusions about causal factors, especially as there was a preponderance of weak study designs, with small numbers of participants and insufficient power to detect small effect sizes [94].

## 13. Perpetuating Factors

As regards perpetuating factors for ME/CFS and outcomes, a systematic review asserted that factors associated with worse prognosis included old age, chronic illness, comorbid psychiatric disorders, and, controversially, belief in a physical cause for the illness [103]. Severity of fatigue and psychiatric morbidity at baseline were associated with persistence twelve months later [104]. Among adolescents, risk factors for prolonged illness include older age at the outset, pain, and poor mental health and self-esteem [105]. Cardiovascular morbidity and mortality are increased in ME/CFS. Oxidative damage to DNA is found both in severe depression and ME/CFS [106], and is also a risk factor for atherosclerosis, hence the increased cardiovascular morbidity in ME/CFS [107]. In addition, reduced coenzyme Q10 may be the cause of chronic heart failure and increased cardiovascular mortality in ME/CFS [108]. In conclusion, it is likely that ME/CFS is attributable to a combination of host and environmental risk factors [109]. In most cases, a number of factors may be involved, of which host factors appear to be most prominent [110]. 

UK study of risk factors for severe ME/CFS (i.e., being housebound or bedbound) found that early management of the illness appeared to be an important determinant of prolonged, severe disease. This observational, questionnaire-based study was designed to identify risk factors for severe (i.e., housebound or bedbound) disease. Exposure to potential risk factors, including familial risks, personality, and early management of the illness, was compared in 124 people with severe disease and 619 mildly ill controls. Severity was determined by self-report and the Barthel (activities of daily living) Index. Premorbid personality was assessed using the Neuroticism and Conscientiousness domains of the International Personality Item Pool (IPIP) scale. Analysis was performed by tests of association and logistic regression. Early management of the illness appeared the most important determinant of severity. Having a mother with ME/CFS was also important. Smoking and personality were not risk factors, neurotic traits being more frequent among the less severely ill. Conscientiousness overall was not related to severity [111]. This confirmed the findings of an earlier population-based study, which showed that shorter illness duration was a significant predictor of sustained remission, and thus early detection of CFS is of utmost importance [112], as well as removal of barriers to healthcare utilisation, which is a serious problem [113].

## 14. Scope for Prevention in ME/CFS

This review has demonstrated that there is little consensus about the nature and impact of risk factors for ME/CFS and, as regards those risk factors about which there is general agreement, few are modifiable. Therefore, there is little scope for programmes of primary prevention, with the exception of organophosphate exposure. 

Secondary prevention is a different matter, however. As detailed above, there are modifiable risk factors for severe and prolonged disease, in particular in mismanagement of the early stages of the illness, including diagnostic delays [111,112] and barriers to healthcare utilisation [113]. Previous work undertaken by the Working Group has considered the reasons for delay in diagnosis, which is a major barrier to healthcare utilisation. We reviewed the literature on knowledge and understanding of ME/CFS among GPs and concluded that between a third and a half of all GPs either disbelieved in the existence of ME/CFS as a genuine clinical entity or had little understanding of it, while a similar proportion of ME/CFS patients expressed dissatisfaction with the primary care that had received, and that these proportions occurred across a wide geographical area and had changed little over many years [114]. We also conducted a survey of how GP knowledge and understanding of ME/CFS was perceived among EUROMENE participants and found that similar misgivings were encountered across Europe [115]. Overall, it appears that, in Europe, a high proportion of GPs, upwards of 50%, do not recognise ME/CFS as a genuine clinical entity and therefore never diagnose it. Among those GPs who do recognise its existence, there is a marked lack of confidence in making the diagnosis and managing the condition. Therefore, estimates of the public health burden of the illness and of its economic impact are likely to be substantial underestimates [7].

Vink and Vink-Niese have demonstrated further scope for secondary prevention within the occupational setting. They demonstrated that patients required to rest at the outset of their illness have the best prognosis and that, on return to work, not pressurising such patients to over-perform could minimise relapses, long-term sick leave, and retirement on medical grounds [116]. Others have pointed out that many ME/CFS patients, particularly the most ill, are neglected by the healthcare system, often due to impediments to diagnosis and associated stigma, and argue for a holistic model of care leading to more supportive interactions between patients and practitioners [117,118].

In children, the experience of an Italian treatment and support initiative in education underlines the importance of early intervention in achieving successful outcomes in ME/CFS [119].

## 15. Associated Features

There may be scope to minimise some of the clinical features of ME/CFS, such as associated orthostatic intolerance, and hence thereby to reduce its economic impact. Thus, cardiovascular symptoms are common in ME/CFS patients. Cardiac dysfunction with low cardiac output due to small left ventricle may contribute to the development of chronic fatigue as a constitutional factor in a considerable number of ME/CFS patients [120], is most marked in patients with orthostatic intolerance [121], and it may be the consequence of a co-morbid hypovolaemic condition [122]. Many ME/CFS patients have a small heart, and this may predispose them to fatigue [123], and to the development of ME/CFS in a well-defined subgroup of ME/CFS patients [124]. A cross-sectional survey found that treatment of orthostatic symptoms in ME/CFS could improve functional capacity and quality of life [125]. Approaches to minimising the impact of orthostatic intolerance include the avoidance of factors that make symptoms worse, including hot surroundings and standing for prolonged periods. Insufficient salt and fluid intake may be a contributory factor to orthostatic intolerance in ME/CFS patients, so should be increased in the absence of contraindications including hypertension, congestive cardiac failure, and renal failure. Support stockings may also help [126]. Pharmacological treatment may help in patients who fail to respond to such conservative measures, including, for example, midodrine, and the mineralocorticoid fludrocortisone [127].

## 16. Conclusions and Recommendations

There is little scope for primary prevention programmes for ME/CFS, because there is little knowledge of, or consensus about, the modifiable risk factors that could be addressed by such a programme. The exception to this is in the use of agrichemicals, particularly organophosphates, where a precautionary principle suggests that Europe-wide programmes of health education to encourage safe use could be beneficial. There is a need for more research on such risk factors for ME/CFS, in order to establish a basis for the development of primary prevention programmes, and there are increasing opportunities for such research to be undertaken. For example, the European Human Biomonitoring programme creates a window opportunity to develop consistent mapping of the distribution of agricultural risk factors, which in turn could enable ecological studies of the distribution of ME/CFS in rural areas [128].

However, by contrast, there is considerable scope for secondary prevention, as improving the management of ME/CFS in the early stages of illness could have an impact in reducing the incidence and prevalence of severe prolonged disease, and thereby also its economic impact. Far too frequently, the primary care management of the illness is characterised by disbelief, lack of knowledge, and misunderstanding. Major benefits could be achieved by improving knowledge and understanding of ME/CFS in general practice, in order to minimise the diagnostic delays that are associated with prolonged illness and increased severity, and hence with increased costs. In addition, further benefits may be achievable through amelioration of associated features such as orthostatic intolerance.

## Data Availability

No new data were created or analysed in this study. Data sharing is not applicable to this article.
